# Re-Thinking Hyperkalaemia Management in Chronic Kidney Disease—Beyond Food Tables and Nutrition Myths: An Evidence-Based Practice Review

**DOI:** 10.3390/nu16010003

**Published:** 2023-12-19

**Authors:** Helen L. MacLaughlin, Erynn McAuley, Jessica Fry, Elissa Pacheco, Natalie Moran, Kate Morgan, Lisa McGuire, Marguerite Conley, David W. Johnson, Sharad K. Ratanjee, Belinda Mason

**Affiliations:** 1 Nutrition Research Collaborative, Department of Dietetics and Foodservices, Royal Brisbane and Women’s Hospital, Herston, QLD 4029, Australiaelissa.pacheco@health.qld.gov.au (E.P.);; 2 School of Exercise & Nutrition Sciences, Queensland University of Technology, Brisbane, QLD 4059, Australia; 3 Nutrition and Dietetics Department, Princess Alexandra Hospital, Woolloongabba, QLD 4102, Australia; 4 Department of Kidney and Transplant Services, Division of Medicine, Princess Alexandra Hospital, Woolloongabba, QLD 4102, Australia; 5 Centre for Health Services Research, The University of Queensland, St. Lucia, QLD 4067, Australia; 6 Kidney Health Service, Royal Brisbane and Women’s Hospital, Herston, QLD 4029, Australia

**Keywords:** chronic kidney disease, potassium, hyperkalaemia, potassium additives, dietary potassium restriction

## Abstract

Potassium dysregulation can be life-threatening. Dietary potassium modification is a management strategy for hyperkalaemia. However, a 2017 review for clinical guidelines found no trials evaluating dietary restriction for managing hyperkalaemia in chronic kidney disease (CKD). Evidence regarding dietary hyperkalaemia management was reviewed and practice recommendations disseminated. A literature search using terms for potassium, hyperkalaemia, and CKD was undertaken from 2018 to October 2022. Researchers extracted data, discussed findings, and formulated practice recommendations. A consumer resource, a clinician education webinar, and workplace education sessions were developed. Eighteen studies were included. Observational studies found no association between dietary and serum potassium in CKD populations. In two studies, 40–60 mmol increases in dietary/supplemental potassium increased serum potassium by 0.2–0.4 mmol/L. No studies examined lowering dietary potassium as a therapeutic treatment for hyperkalaemia. Healthy dietary patterns were associated with improved outcomes and may predict lower serum potassium, as dietary co-factors may support potassium shifts intracellularly, and increase excretion through the bowel. The resource recommended limiting potassium additives, large servings of meat and milk, and including high-fibre foods: wholegrains, fruits, and vegetables. In seven months, the resource received > 3300 views and the webinar > 290 views. This review highlights the need for prompt review of consumer resources, hospital diets, and health professionals’ knowledge.

## 1. Introduction

Hyperkalaemia is rare in the general population, but the risk increases with declining kidney function as kidney mechanisms for potassium clearance are compromised. Hyperkalaemia is associated with increased risks of cardiac arrythmia, hospitalisation, and death in those with reduced kidney function [1]. The potential for potassium toxicity has been extrapolated into widespread recommendations to limit foods high in potassium to prevent or manage hyperkalaemia in people with chronic kidney disease (CKD). Early observational studies examined the toxicity of potassium salts and demonstrated that high doses of supplemental potassium led to hyperkalaemia and cardiac rhythm changes in people with kidney damage [2,3]. It was noted in these studies that the potassium salts were widely distributed into intracellular compartments, and excretion in urine continued until equilibrium was reached [2,3].

Previous clinical practice guidelines suggested a cautious approach to including potassium-rich foods in the diets of people with CKD to limit the risk of harm, with little supporting evidence for this practice [4,5,6]. Earlier European guidelines suggested dietary restriction if serum potassium was >6.0 mmol/L, and that other causes should also be investigated [5]. Elsewhere in the document, description of patients on a potassium-restricted diet may have inadvertently reinforced potassium restriction as a requirement, rather than a treatment, for hyperkalaemia. Current international evidence-based practice guidelines suggest that it is reasonable to adjust dietary potassium intake to maintain serum potassium within the normal range, although this statement is based on expert opinion due to a lack of available evidence from dietary intervention studies [7]. In 2020, the Kidney Disease: Improving Global Outcomes (KDIGO) controversies conference report on management of dyskalaemia in kidney diseases recognised the poor evidence base to support dietary restriction of potassium to manage hyperkalaemia, together with the potential for harm by restricting diet quality and nutrients beneficial to health in the absence of hyperkalaemia [8].

The practice of recommending restriction of fruit and vegetable intake to manage hyperkalaemia in people with CKD is common, and widely disseminated in patient education materials [9]. There is a growing evidence base supporting healthy dietary patterns for improving CKD outcomes and reducing mortality [10,11,12,13]. Healthy dietary patterns include the Mediterranean diet pattern and the Dietary Approaches to Stop Hypertension (DASH) dietary pattern which are inherently higher in fruits, vegetables, nuts, seeds, and wholegrains and lower in meat, milk, and processed foods.. These dietary patterns are high in potassium, which can create a conflict in health messaging for people with CKD. Potassium in plant sources is less available for digestion and absorption than potassium from animal foods and food additives, due to the integrity of the cellular structure in plant cell walls [14]. This juxtaposition prompted a review of potassium homeostasis and the effect of dietary potassium on serum potassium, which sought to determine if any new evidence was available to guide dietetic practice in the management of hyperkalaemia in CKD.

Mechanisms of potassium regulation have been well reviewed by others [15] so will be briefly discussed here. Potassium is primarily an intracellular cation and is tightly regulated via signalling pathways which increase uptake of potassium by the liver and skeletal muscle and stimulate increased excretion via kidney tubules [16]. After meals, these processes maintain stable extracellular plasma potassium, activated by gut signalling via brain, pancreas, muscle, adrenal, and kidney mechanisms [17]. Consumption of carbohydrate with a potassium-containing meal activates pancreatic insulin release which in turn activates sodium potassium adenosine triphosphatase (ATPase) [16]. Gut–brain pathways detect potassium and stimulate aldosterone production and increase kidney clearance of potassium via distal nephron sodium channels and the renin–angiotensin–aldosterone system (RAAS) [18]. Potassium is also excreted through the colon, which accounts for a greater proportion of potassium removal as kidney function declines [8].

Several mechanisms can disrupt this homeostasis. Diabetes or beta-blockade-associated hyporeninemic hypoaldosteronism can be common causes of hyperkalaemia when kidney function and dietary intake of potassium do not appear to be contributory causes [19]. Nonselective beta blockers interfere with potassium uptake by cells via beta-2 adrenergic mediated pathways [20]. Meals containing mainly animal proteins, which are net acid producing, or a state of metabolic acidosis, result in increased free hydrogen ions, which causes potassium to shift from intracellular to extracellular spaces. The set of randomised controlled trials by Goraya et al. demonstrated that base-producing fruits and vegetables decreased metabolic acidosis in CKD stages 3 and 4 as effectively as dosing with sodium bicarbonate [21,22]. A low potassium diet can lead to an adaptation to conserve potassium by reducing the number of ATPase channels in muscle and increasing potassium retention via kidney tubules [23], which may contribute to acute dietary-induced hyperkalaemia when potassium intake increases. Exercise can induce hyperkalaemia with a single intense bout, and some studies show that exercise training can dampen the exercise-induced rise in blood potassium [24]. Therefore, an acute increase in dietary potassium, acidosis, insulin resistance and hyperglycaemia, acute exercise or muscle catabolism, constipation, or the introduction of a RAAS inhibitor are all potential contributors to hyperkalaemia in the setting of CKD, and diet plays a contributory role to many of these states.

This evidence-based practice review sought to synthesise studies examining associations between dietary potassium intake and outcomes, including hyperkalaemia, in people with CKD since the systematic evidence review for the KDIGO controversies conference was completed in 2017 [8]. The aim of this review is to inform revision of education resources for use by dietitians, physicians, nurses, general practitioners, and consumers for the management of hyperkalaemia in people with CKD.

## 2. Materials and Methods

A pragmatic literature search was undertaken using MEDLINE for studies published from 2018 to October 2022. Key title/abstract search terms were “potassium” OR “hyperkal*” and “kidney OR renal”. Included studies examined the relationship between dietary potassium and serum potassium, hyperkalaemic events, dietary treatment of hyperkalaemia, or progression of kidney disease in people with CKD stages 1–5 including dialysis. Accepted study designs were systematic reviews, observational cohort studies, randomised controlled trials (RCTs), and uncontrolled trials. Individual case studies, case series, and studies on efficacy of potassium binders were excluded. No language limitation was applied. In addition, we also reviewed studies examining the bioavailability of potassium in fresh, cooked, and processed foods to determine how food preparation and processing can affect potassium content of consumed foods.

Studies were collaboratively reviewed by a team of dietitians with a range of experience (1–20 years) as practising dietitians in kidney nutrition (B.M., E.M., K.M., L.M., H.M., N.M., J.F.). Monthly meetings were held from January to October 2022 to review studies. Discussions focused on the interpretation of study findings to make recommendations on dietary management of hyperkalaemia in clinical settings and for developing a consumer education resource. Two summary tables were prepared by one author (H.M.) and independently checked by a second author (M.C.). First author, year of publication, study design, findings, discussion points, and practice implications for each study were recorded in both tables.

Fortnightly meetings were held from November to December 2022 to translate the evidence from the studies into the consumer resource, using the standard Nutrition Education Materials Online (NEMO) template for consumer resources (https://www.health.qld.gov.au/nutrition/about-us). Ideas were workshopped and discussed, and consensus reached by reviewing the available evidence in the summary of studies. In cases where the group could not agree, the original studies were reviewed again, and consensus agreement was reached on the interpretation of the findings through further discussion. Where direct evidence for recommendations was lacking, consensus expert opinion was reached within the group, based on the interpretation of the available evidence and clinical expertise.

The draft resource was refined over several iterations and reviewed for readability and comprehension by seven consumers with lived experience of CKD. Their feedback was incorporated into revised drafts. Further consultation and review were undertaken with the Dietitians Australia, Queensland Kidney Interest Group—a voluntary collective of dietitians delivering nutrition care in hospitals and health services in Queensland to people with CKD in conjunction with nephrologists—for expert review of content, presentation, and useability, through both a workshop and circulation of the draft resource to all members of the group for comment. The final step of the editing process was a review by the NEMO editorial team to ensure that a robust evidence review and consultation process had been followed. In addition to the development of the consumer resource, the authors prepared a 30 min webinar presentation of the evidence review to support broad dissemination and training for clinicians.

## 3. Results

### 3.1. Review of the Evidence

Eighteen studies were included in the evidence review, and key findings are summarised in Table 1 for clinical studies and Table 2 for food preparation and processing. There were four systematic reviews [7,8,25,26], eight observational studies [10,27,28,29,30,31,32,33], one RCT [34], and five studies on food processing or preparation methods [35,36,37,38,39].

The 2016 evidence review for the 2020 Kidney Disease Outcomes Quality Initiative (KDOQI) Nutrition in CKD guideline update found no clinical trials directly examining the relationship between dietary potassium consumption and either serum levels or clinical outcomes [7]. The guidelines suggested that dietary adjustments of potassium intake were a reasonable intervention to maintain serum potassium in the normal range. The KDIGO report examined evidence addressing the relationship between potassium intake, mortality and CKD incidence, progression, initiation of dialysis, or kidney graft failure [8]. Across 10 prospective observational studies, surrogates of high potassium intake (mostly 24 h urinary potassium) were associated with a lower risk of CKD incidence and progression [8]. It was unclear whether this was explained by potassium intake per se or whether potassium was a proxy for higher plant consumption or a specific eating pattern. Studies did not report potassium levels or incidence of hyperkalaemia. Three studies examined diets high in fruits and vegetables in CKD and measured the change in serum potassium as a safety outcome [22,40,41]. All studies reported no change in serum potassium with a diet rich in fruits and vegetables, and there were no adverse hyperkalaemic events. Overall, there was not sufficient evidence at that time to recommend liberalisation of dietary potassium intake, and dietary potassium restriction as a strategy to manage acute hyperkalaemia was still recommended. However, the report acknowledged that generalised restriction to avoid hyperkalaemia may unnecessarily limit people with CKD in receiving the beneficial effects of potassium-rich diets [8].

Two more recent systematic reviews also examined the effect of dietary potassium on CKD progression [25,26]. In CKD stages 1–2, higher potassium intake is suggestive of lower risk of CKD progression; however, in CKD stages 3–5 pre-dialysis, data are mixed [26]. Studies mostly estimated dietary potassium from urinary potassium, and overall potassium intake was lower than the guideline recommendation [26]. The second review examined stage 3 CKD populations and found no association between potassium intake and CKD progression or mortality [25]. Two small randomised trials provided very low-quality evidence for potassium restriction lowering serum potassium by 0.22 mmol/L [42,43]. There was contamination of the intervention in one study by the use of the potassium lowering drug, sodium polystyrene sulfonate, as dietary restriction alone did not achieve the target serum potassium level of 4.5 mEq/L [42]. The other study compared the effect of two liquid oral nutrition supplements as the sole source of nutrition, so it is not transferable to a food-based approach [43]. These findings indicate a small clinical effect but are not transferable to food-based interventions or recommendations.

Five observational studies published since these reviews examined associations between dietary and serum potassium and demonstrated no association between total dietary potassium and serum potassium in CKD, including haemodialysis populations [27,28,29,31,32]. Hyperkalaemia incidence was not associated with total dietary potassium intake [27,28]. Higher intake of fruits and vegetables or plant-based diets was not associated with serum potassium or hyperkalaemia [27,29]. Other benefits associated with dietary patterns inherently high in fruit and vegetables and potassium are slower progression of CKD and a reduction in all-cause mortality in people with CKD stages 2–4 over a median follow-up period of seven years [10]. Lower dietary potassium intake was associated with increased all-cause mortality in a cohort of haemodialysis patients, and the association remained after adjustment for potential confounders [30]. In a large cohort of haemodialysis patients randomly selected from 37 dialysis centres, higher adherence to the DASH dietary pattern predicted lower serum potassium, and individual foods positively correlated with serum potassium were milk, eggs, beef, pork, chicken liver, fatty fish, squid, octopus, banana, canned fruit, wine, and coffee [31]. One study in children across stages 1–5 CKD reported that dietary fibre was positively associated with dietary potassium, that dietary potassium was not associated with serum potassium, and that restriction of higher potassium foods may limit fibre intake [32]. Observational studies may have methodological limitations including the known limitations of dietary data collection. Non-dietary factors associated with hyperkalaemia included diabetes, lower serum bicarbonate [27], and previous hyperkalaemia [29].

Two studies examined the effect of increasing potassium intake on serum potassium, with 40 mmol of potassium chloride [33] or an additional 60 mmol of potassium obtained from food [34] raising serum potassium by 0.4 mmol/L and 0.2 mmol/L, respectively. The former study was undertaken in patients with CKD stages G3b-4 during the run-in phase for a trial to assess the long-term effect of potassium supplementation on kidney function [33]. Most patients did not experience hyperkalaemia, even with concurrent use of RAAS inhibitors. Hyperkalaemia incidence was 11% and was associated with older age and higher baseline potassium, although higher baseline potassium was also associated with smaller increases in serum potassium with supplementation [33]. The 60 mmol increase in potassium in the second study was achieved with a DASH-style dietary pattern, compared to a standard low potassium diet in CKD stage 3, and diets were matched for macronutrients, sodium, and phosphorus [34]. The slight increase in serum potassium with a large increase in dietary potassium indicated that plant sources of potassium were less bioavailable than supplemental potassium salts. In both studies, patients with a history of hyperkalaemia were excluded. Potassium balance studies suggest that the bioavailability of potassium is lower in plant foods than foods of animal origin [14]. Diets high in potassium from intact fruit and vegetable sources result in lower urinary potassium recovery than diets high in processed and animal sources of potassium, as up to 40% of the potassium is retained in cell walls during digestion and not absorbed [14].

Studies of food preparation techniques demonstrate that soaking, hot soaking, and cooking in water (steaming, microwaving, boiling, double boiling, and pressure cooking) all significantly reduce potassium content in meats, legumes, vegetables, and tubers [36,37,39]. Methods for studying reductions in potassium content with different food preparation techniques differ widely and there is no standardised technique [36]. Fibre, found only in plant foods, may be an additional factor to reduce the availability of potassium in fruits, vegetables, seeds, and wholegrains. Faster transit time due to higher fibre content of meals may reduce constipation risk, which is also a known risk factor for hyperkalaemia [37]. Additionally, potassium in plant foods is only 50–60% bioavailable for digestion and absorption as the potassium is contained within cell walls, and thus, a significant amount passes through undigested and is excreted in faeces [14]. Dietary studies measuring urinary potassium excretion consistently demonstrate the lower bioavailability of potassium in diets high in fruits and vegetables, including the DASH dietary pattern compared to diets higher in meats, processed meats and lower in vegetables [38]. Conversely, potassium not bound in cell walls, such as in potassium salts/additives or from animal sources, is 95–100% absorbed during digestion [14,38].

Potassium additives are found in a wide variety of processed foods, with processed meats, bakery products, non-alcoholic beverages, sauces, processed cheese, and ready-to-eat foods containing the highest amounts [35,37]. Up to 38% of processed foods in Europe contain potassium additive, with potassium sorbate (E202) and potassium phosphate (E340) both commonly added to foods during processing, including as a sodium replacement to lower salt content [35,38].

### 3.2. Dissemination of Findings

This review concluded that there was little evidence to support overt dietary restriction of fruits and vegetables in the prevention and management of hyperkalaemia. The education resource was developed with a shift in focus to investigate non-dietary causes of hyperkalaemia initially, followed by a recommendation to reduce excess intake of non-plant sources of potassium, such as potassium additives and excessive servings of milk products, meats, and low-nutritional-value potassium containing snack foods such as potato chips (crisps), chocolate, and processed meat products. Fruit and vegetable intake is based on the national dietary guideline recommendations to include five servings (75 g per serve) of vegetables and two servings of fruit (150 g per serve) every day [44] but not more, and it recognises the growing support for plant-predominant diets in people with CKD [12,45,46]. Lists of lower and higher potassium-containing fruits or vegetables were intentionally omitted due to consideration of lower bioavailability across all fruits and vegetables, and a lack of evidence supporting this as a management strategy for hyperkalaemia. A pictorial food swap guide suggested switching from processed to freshly prepared foods, reducing meat portions, and replacing processed meats with nuts, seeds, and fish or pulses, swapping potassium-containing drinks for plain water, nut or oat milks, and lower-potassium snack alternatives. The education resource was published on the Queensland Health NEMO website in January 2023 and is available for download (https://www.health.qld.gov.au/__data/assets/pdf_file/0021/1206471/renal-k-high.pdf).

The 30 min webinar presentation of the evidence review was initially delivered to the Dietitians Australia, Queensland Kidney Interest Group in December 2022. This presentation provided the supporting information required to critically review the new patient education resource and had the dual purpose of providing a continuing professional development opportunity for dietitians practising in the field of kidney dietetics. Additionally, a recording of the webinar was made available online (https://vimeo.com/797179097) to support the further dissemination of the evidence review and upskilling of dietitians, nephrologists, and other members of the multidisciplinary team. All three local universities teaching dietetics programs were provided with the webinar link to update teaching practice. Further dissemination occurred through local and national conference presentations and training sessions to over 300 health professionals. The resource was accessed >3300 times between February and August 2023, which was a 63% increase in views compared to the same period in 2022 for the previous version of the resource. The webinar was viewed 290 times from January to September 2023.

## 4. Discussion

This evidence review challenges the status quo and supports a change in the practice of dietary management of hyperkalaemia. There were no trials investigating the effect of restricting fruit and vegetable intake to treat or prevent hyperkalaemia, despite widespread recommendation in clinical practice. Over the last decade or so, studies have emerged which suggest that dietary potassium intake is only weakly associated with serum potassium in people with CKD [8,25,28,32,34], and dietary potassium from animal sources, highly processed foods, and additives is likely to have a greater impact on serum potassium than potassium from minimally processed plant sources [14,33,34]. This strongly challenges practices limiting potassium intake from fruits and vegetables to reduce the risk of, and treat, hyperkalaemia. Fruits, vegetables, dairy foods, and meat are common sources of dietary potassium in whole foods. Lower bioavailability of potassium and higher fibre in fruits and vegetables may explain the lack of association between fruit and vegetable intake and serum potassium in observational studies. Higher intake of meat, milk, and processed foods may by a key contributor to chronic hyperkalaemia due to higher bioavailability and large portions [14,38]. Emerging evidence suggests that diets higher in plant foods, including fruits and vegetables, may be protective against kidney disease progression and mortality in kidney failure [10,28]. However, only 28% of patient resources recommend reducing foods with potassium additives, and 100% suggest reductions in fruit and vegetable intake [9].

Studies have demonstrated that medications including RAAS inhibitors, diabetes, plasma bicarbonate, plasma creatinine, and constipation are all associated with hyperkalaemia risk [8]. Given the weak association between diet and serum potassium, other potential causes of hyperkalaemia should be investigated and addressed prior to implementing dietary restrictions. Additionally, people with CKD report a poorer quality of life when dietary restrictions are imposed [47], further demonstrating the importance of eliminating unnecessary or excessive dietary restrictions not supported by evidence for patient benefit. Several dietary mechanisms support the plausibility of higher intake of fruits and vegetables and lower hyperkalaemia risk, even in CKD when clearance is reduced. Co-consumption of carbohydrate can support potassium uptake into cells through insulin-stimulating mechanisms to shift potassium intracellularly through ATPase channels, net base-producing fruits and vegetables decrease metabolic acidosis and the subsequent shift of potassium extracellularly to buffer the increase in hydrogen ion concentration, and fibre promotes reduced transit time and higher colonic excretion of potassium [15,48,49]. 

Optimal amounts of fruits and vegetables in the diets of people with CKD remain ill defined, and while current evidence suggests that it is safe to recommend two servings of fresh fruit and up to 375 g of vegetable servings per day in accordance with national dietary guidelines, but not higher, we await further studies to verify this. Therefore, caution is still warranted and unlimited fruit and vegetable consumption is not recommended in practice. Furthermore, swapping concentrated forms for fresh options, limiting root vegetable consumption to one serving per day, and utilising cooking and food preparation methods to reduce potassium content are suggested in the new education resource to ensure safe consumption.

In the past, food composition tables of potassium content were often given to patients, and we no longer support this approach as it fails to consider bioavailability, cooking methods, portion sizes, combinations of foods, fibre, dietary acid load, and individual risk factors such as diabetes and serum creatinine. Food composition tables require complex interpretation by an expert clinician to take these contributing factors into account. Without consideration of portions consumed, bioavailability, and fibre, people with CKD may over-restrict consumption of fruit, vegetables, legumes, nuts, and wholegrains, resulting in their opting for undesirable food choices. Dietitians are well positioned to assess the root cause of hyperkalaemia and offer tailored dietary recommendations for management of hyperkalaemia which considers contributing factors, which may include excess intake of higher potassium fruits and vegetables in some patients. Recommendations need to differentiate between different sources of potassium and bioavailability rather than only considering total dietary potassium consumed. In addition, the whole dietary pattern needs to be considered in recommendations to reduce conflicts in health messaging, especially regarding fruit and vegetable intake.

Our evidence-based practice review has some limitations, mostly based on the quality of studies available for critical appraisal. Intervention studies excluded people with a history of hyperkalaemia [33,34] so their findings cannot be extrapolated to those at higher risk. Observational studies are limited by the quality of their data collection methods, and dietary data have known limitations [50]. However, the studies are consistent in their findings, and none have demonstrated an association between higher intake of potassium from food and hyperkalaemia. Caution is still warranted for dietary recommendations in CKD stage 5 non-dialysis and in those with a history of hyperkalaemia, as there remains an evidence gap for these higher-risk patients. Finally, our study methods did not include a full systematic review of the evidence, so we may have inadvertently missed studies related to our topic.

There is an urgent need for more research, particularly for well-designed trials examining the impact of the DASH dietary pattern, Mediterranean-style diets, and other plant-based dietary patterns, designed to examine potassium-specific pre-specified outcomes, along with measures of CKD progression and patient-reported outcomes. Such trials need to maintain optimal RAAS inhibition, given the recent findings demonstrating poorer outcomes in those with a reduction or cessation of RAAS inhibition therapy after an episode of hyperkalaemia [51]. Interventions could also be considered that combine diet and exercise, to examine the impact of increasing skeletal muscle to increase potassium buffering capacity through ATPase channels and potentially increase post-prandial potassium storage capacity. A platform trial design or use of a root cause analysis tool may support more rapid development of evidence and translation into clinical practice.

## 5. Conclusions

In conclusion, potassium management in CKD is multifactorial, and an individual assessment is needed of the non-dietary causes and potential dietary contributors, including potassium additives. Existing studies are largely observational, and intervention studies excluded those with a history of hyperkalaemia. Dietary modifications may be considered when other causes have been excluded or managed, and the focus should be on reducing potassium from additives and animal sources, due to the high bioavailability, and introducing a healthy dietary pattern. There is an absence of evidence to support the practice of limiting fruit and vegetable intake based on food composition tables without consideration of other causes of hyperkalaemia, bioavailability of potassium, and total fibre intake. Management of hyperkalaemia may encourage a healthy dietary pattern, with sufficient fibre in the diet to support increased faecal excretion of potassium and adequate but not excessive fruit and vegetable servings, with ongoing adjustment of dietary intake with potassium monitoring as clinically indicated. Further research is required to examine the safety of a healthy dietary pattern in populations which include those at high risk of a recurrent episode of hyperkalaemia.

## Figures and Tables

**Table 1:** Summary of studies published between 2018 and 2022 relating to hyperkalaemia in people with chronic kidney disease.

Author and Year	Study Design	Findings	Discussion	Practice Implications
Clase et al., for KDIGO group, 2020 [8]	Systematic review, completed 2018.	Observational studies suggest that surrogates of high potassium intake are associated with lower risk of CKD incidence and progression.	Well-designed studies on strategies to manage hyperkalaemia are lacking.	Poor evidence to support dietary potassium restriction. Routine low potassium diets in CKD may deprive people of other beneficial effects of potassium rich diets.
Ikizler et al., for KDOQI guidelines 2020 [7]	Systematic review, completed 2016.	No clinical trials were identified that directly examined the relationship between dietary potassium intake and serum levels or clinical outcomes.	Authors emphasise that medications, kidney function, hydration status, acid–base balance, glycaemic control, catabolic state, vomiting, diarrhoea, constipation, and gastrointestinal bleeding can also influence serum potassium levels.	All factors should be considered when devising treatment strategies to establish and maintain normokalaemia. Dietary restrictions should consider the overall diet composition. Treatment of hyperkalaemia should first address potential non-dietary contributors.
Morris et al., 2022 [25]	Systematic review, completed 2018.	Two RCTs were included—very low-quality evidence due to risk of bias. Dietary potassium restriction (weighted mean 1295 mg/day) compared to no restriction (weighted mean 1570 mg/day) reduced serum potassium by 0.22 mEq/L, although potassium binders were used in one study [18]. No association between dietary potassium intake and CKD progression or death in observational studies.	Dietary potassium source was oral nutritional supplements in one trial; in the other trial, half the patients in the restricted group required potassium binder medication to achieve the goal serum potassium level; dietary potassium was extrapolated from urinary potassium excretion.	The evidence supporting dietary potassium restriction in CKD is very low quality. Cannot extrapolate results from these studies to food-only interventions; only 50% of participants responded to dietary potassium restriction and the clinical effect was small.
Picard et al., 2020 [26]	Systematic review, completed 2019.	No intervention trials on dietary potassium intake and CKD progression. In early CKD, six out of nine observational studies demonstrated a relationship between high potassium intake and lower risk of CKD progression, or low potassium intake and a higher risk of CKD progression. In stages 3 to 5 CKD observational studies, results were equivocal. Four studies examined dietary and serum potassium. No studies reported a higher risk of death with higher potassium intake.	Potassium intake appears to have a protective effect on CKD progression. Overall potassium intake was low. In the four studies which examined serum potassium or hyperkalaemia events across quartiles of potassium intake, there were no associations between potassium intake, serum potassium, or hyperkalaemia	Preliminary studies suggest that the risk of hyperkalaemia with a high potassium diet may be low, although well-designed intervention trials are needed.
Hu et al., 2020 [10]	Prospective observational cohort study using the CRIC data (eGFR 20–70 mL/min/ 1.73 m^2^), diet assessed by FFQ and scores for 4 diet quality indices calculated.	Lower risk of CKD progression and all-cause mortality was associated with higher scores for the Alternate Healthy Eating Index-2010, the alternate Mediterranean Diet, and DASH diet scores. Higher Healthy Eating Index-2015 scores were predictive of lower risk of all-cause mortality but not CKD progression.	Participants with high scores for diet quality had higher total potassium intake and lower dietary acid load across all indices of diet quality. Vegetables and nuts were the protective components of the Mediterranean diet pattern for lower likelihood of CKD progression.	Diets high in fruits, vegetables, nuts, and legumes may be beneficial for kidney function, supporting a shift away from single nutrient management to consideration of food-based dietary patterns.
Ramos et al., 2021 [27]	Observational, cross-sectional study in 2 groups: (a) stages 3–5 CKD at first referral to dietitian, and (b) prevalent haemodialysis patients; diet assessed from 3-day food records.	No association between dietary potassium and serum potassium or hyperkalaemia in either group; hyperkalaemia was associated with diabetes and lower serum bicarbonate in CKD stages 3–5, and with diabetes and serum creatinine in the HD group. Fruit and vegetable intake was not associated with serum potassium or hyperkalaemia.	Dietary potassium intake is not the main determinant of serum potassium.	Consider animal, plant, and additive sources of dietary potassium, as bioavailability and effect on serum potassium may differ between sources.
Bernier-Jean et al., 2021 [28]	Sub-analysis of the DIET-HD prospective study in a cohort of haemodialysis patients, diet assessed by FFQ.	No association between dietary potassium and serum potassium, or hyperkalaemia or death; higher serum potassium was associated with increased risk of cardiovascular death; incidence of hyperkalaemia was the same across all quartiles of potassium intake.	Potassium additives in processed foods were not assessed in the dietary intake and are more easily absorbed than potassium from food sources. Potassium additives in processed foods were not assessed in the dietary intake and are more easily absorbed than potassium from food sources.	Less restriction of fruits and vegetables may provide health benefits and reduce the burden of dietary modification; non-fruit-and-vegetable-focused interventions to reduce serum potassium should be considered.
Gonzalez-Ortiz et al., 2021 [29]	Observational, longitudinal study in single-centre haemodialysis cohort in Mexico, diet assessed by 3-day food record and dietitian interview every 4 months.	No association between higher adherence to plant-based diets and serum potassium or hyperkalaemia; higher plant-based eating scores were associated with an 11% increase in risk for not achieving target protein intake of >1.1 g/kg/day (95% CI 1.04–1.19), protein intake with higher plant-based eating was 0.9 g/kg/day and 1.1 g/kg/day with lower plant-based eating.	While no association between higher intake of plant foods and hyperkalaemia was observed, individualised dietary counselling is advised due to differences in responses between individuals including prior frequency of pre-dialysis hyperkalaemia.	A dietary pattern higher in plant foods and lower in animal foods may be suitable for haemodialysis; individualised counselling is recommended and should consider nutritional status, constipation, previous hyperkalaemia, and dialysis adequacy.
Narasaki et al., 2021 [30]	Observational cohort study of 415 patients in 16 haemodialysis centres in California, diet assessed by FFQ.	Lower dietary potassium intake was associated with higher risk of death (HR 2.65, 95% CI 1.40–5.04) in fully adjusted model comparing lowest tertile of intake to highest tertile.	Dietary restriction of potassium may reduce total nutrient intake and increase risk of malnutrition, and/or increase risk of cardiovascular disease due to restriction of cardio-protective foods and nutrients.	Excessive dietary potassium restriction in haemodialysis patients may be harmful; further research is needed to define optimal potassium intake and dietary sources.
Garagarza et al., 2022 [31]	Observational, cross-sectional, multicentre study in randomly selected haemodialysis patients from 37 dialysis centres, diet assessed by FFQ.	No association between dietary potassium and serum potassium. Higher adherence to the DASH dietary pattern (DASH diet score) was a predictor of lower serum potassium in the adjusted model; foods that showed a positive correlation with serum potassium were milk, eggs, beef, pork, chicken liver, fatty fish, squid, octopus, banana, canned fruit, wine, coffee.	Patient population all dialysed using online haemodiafiltration technique; diet assessment undertaken by dietitian during interview with participants. The DASH diet pattern is high in potassium and low in sodium.	Different sources of potassium (animal, plant, additive) may contribute unequally to hyperkalaemia because of bioavailability. A dietary pattern high in plant-based foods, fibre, and carbohydrates may be beneficial for controlling serum potassium level in haemodialysis.
El Amouri et al., 2022 [32]	Prospective observational multicentre study in children with CKD stages 1–5 excluding dialysis, diet assessed by 3-day food record and dietitian interview every four months, 24 h recall used if no 3-day record completed.	No association between dietary potassium intake and serum potassium; positive association between fibre intake and dietary potassium; no association between dietary fibre and serum potassium.	Excessive restriction of high potassium foods limits fibre intake; meat and meat products often contain as much or more potassium per serving than fruit and vegetables and can be overlooked as a source of dietary potassium. Plant sources of potassium enhance intracellular uptake of potassium as they are alkaline and stimulate insulin production, in addition to being less bioavailable.	Consider non-dietary causes, then low-nutritional-value high-potassium foods and foods with potassium additives, then address cooking methods and prioritise high-fibre plant foods.
Gritter et al., 2022 [33]	Single-arm run-in phase for placebo controlled RCT to observe changes in plasma potassium with short-term potassium chloride supplementation in stages G3b-4 CKD.	A daily dose of 40 mmol potassium led to an increase in plasma potassium of 0.4 mmol/L (from 4.3 mmol/L to 4.7 mmol/L); higher baseline plasma potassium and diuretic use were associated with a smaller increase in potassium with supplementation; hyperkalaemia incidence was 11% [21/191] and was associated with older age and higher baseline potassium.	The effect of potassium supplementation on serum or plasma potassium is likely higher than the effect from food as the bioavailability from foods is much lower.	Potassium from additives and supplements has a greater impact on blood levels of potassium than potassium from foods, due to the higher bioavailability.
Turban et al., 2021 [34]	Double-blinded randomised two-period crossover feeding trial to determine safety of higher (100 mg) versus lower (40 mg) potassium intake in adults with stage 3 CKD. Diets matched for macronutrients, sodium, and phosphorus.	Higher potassium diet (100 mmol/day) increased serum potassium by 0.21 mmol/L after four weeks compared to the lower potassium diet (40 mmol/d) (*p* = 0.003). Hyperkalaemia (serum K^+^ 5.5 mmol/L) occurred in both the higher and lower potassium diets and was more likely during the higher potassium diet [odds ratio 2.5, 95% CI 1.04 to 6.00]. Confirmed hyperkalaemia in two participants—both had known risk factors (history of hyperkalaemia and use of dual RAAS blockade).	A large increase in dietary potassium caused a small rise in serum potassium during the study. Confirmed hyperkalaemic events are more likely in those with known non-dietary risk factors.	Individualised management of dietary potassium is indicated. Assessment of hyperkalaemia history, medications, and glycaemic control can be integrated with dietary assessment.

Abbreviations: CKD, chronic kidney disease; CRIC, Chronic Renal Insufficiency Cohort; DASH, Dietary Approaches to Stop Hypertension; HR, hazard ratio; KDIGO, Kidney Disease Improving Global Outcomes; KDOQI, Kidney Disease Outcomes Quality Initiative; RAAS, renin–angiotensin–aldosterone system; RCT, randomised controlled trial; FFQ, food frequency questionnaire.

**Table 2:** Summary of studies published between 2018 and 2022 examining potassium in food preparation and food processing.

Author and Year	Study Design	Findings	Discussion	Practice Implications
Batista et al., 2021 [36]	Systematic review of potassium reduction by food preparation techniques.	Cooking in water (saucepan, microwave, steaming, oven, pressure cooker) significantly reduces the potassium content in legumes, vegetables (including tubers and root vegetables), cereals and grains, meats, and fruits. Dry-heat cooking and soaking also reduce potassium content to a smaller extent.	No studies evaluated food preparation techniques for processed foods containing potassium additives; study methods varied considerably and there was no standard method to analyse samples.	Soaking and then cooking legumes/pulses in water will significantly reduce the potassium content; all cooking methods will reduce the potassium content of most foods.
Cupisti et al., 2018 [37]	Narrative review on potassium and fibre and effect of food processing.	Food preparation techniques can remove up to 60–80% of the potassium content of some raw foods. Boiling and double boiling reduced potassium content more than soaking for vegetables and fruit. Cubing or shredding before boiling further reduced potassium content in potatoes. Potassium additives are found in preserved meats, sauces, processed cheese, ready-made stuffed pasta, wine, and some packaged foods with a long shelf life.	Dietary interventions are complex, but recommendations must be simple and easy to implement; use traffic-light-type colour-coded system for lower, moderate, and higher potassium content foods; avoid foods containing potassium additives.	Choose foods with higher fibre content and lower net acid load to achieve potassium reduction while maintaining fibre intake and avoiding excess potassium intake.
de Abreu et al., 2022 [39]	Observational study of the impact of hot-water soaking on potassium content of foods.	Soaking chopped foods in just-boiled deionised water for 5–10 min reduces potassium content by 30–50% for meats, 30–40% for vegetables, 10–20% for tubers, and 40% for legumes and grains.	A short period of soaking in hot water is a practical method to reduce the potassium content in the fresh foods tested. The reduction in potassium seen in the study may be lower if tap water is used and only a limited range of foods were tested.	Meats, vegetables, and legumes can be soaked in water to reduce the potassium content prior to use in cooking. Short soaking does not limit the way foods can then be prepared.
Picard et al., 2019 [38]	Narrative review on potassium bioavailability.	Potassium bioavailability from fruits and vegetables has been estimated to be 50–60%. Higher fruit and vegetable intake in CKD can improve blood pressure and reduce metabolic acidosis. Potassium bioavailability from food additives is 95–100% and may be added to foods containing no natural potassium to lower sodium content.	Dietitians and nutrition guidelines should consider potassium additives in potassium intake guidelines and education resources.	Implementing potassium restrictions is best conducted by dietitians as they balance restriction with meeting nutritional requirements and consider the whole diet.
Martínez-Pineda et al., 2021 [35]	Cross-sectional study of food additives in processed foods in Europe.	37.6% of 715 labelled food products contained potassium additives; processed meats, bakery products, non-alcoholic beverages, and ready-to-eat foods contained the highest amounts.	Different countries use different additives; potassium sorbate (E202) and potassium phosphates (E340) are commonly used.	Potassium additives are widely used in processed foods. Education for hyperkalaemia management in CKD should include the high prevalence of potassium additives in foods.

## Data Availability

Data are contained within the article and linked materials.
